# A rare case of gastric ascariasis

**DOI:** 10.1128/asmcr.00163-25

**Published:** 2026-03-16

**Authors:** Sharon Pan, Kevin Yu, Blaine A. Mathison, Yan Gao, Marc Roger Couturier, Salem Muayad, Violeta Chávez, Ranya Selim

**Affiliations:** 1 Department of Internal Medicine, The University of Texas Health Science Center at Houston 12340, Houston, Texas, USA; 2 Division of Gastroenterology and Hepatology, Department of Internal Medicine, The University of Texas Health Science Center at Houston 12340, Houston, Texas, USA; 3 Department of Pathology, University of Utah 7060, Salt Lake City, Utah, USA; 4 ARUP Laboratories 33294, Salt Lake City, Utah, USA; 5 Department of Pathology and Laboratory Medicine, McGovern Medical School, University of Texas Health Science Center at Houston 12340, Houston, Texas, USA; 6 NorDx, MaineHealth, Scarborough, Maine, USA; Pattern Bioscience, Austin, Texas, USA

**Keywords:** ascariasis, gastric, case report, *Ascaris*

## Abstract

**Background:**

Ascariasis, caused by the parasitic roundworm *Ascaris lumbricoides*, is one of the most common helminthic infections globally. Spread by contaminated soil through the fecal–oral route, *A. lumbricoides* typically infects the small intestine and has rarely been reported to infect the stomach.

**Case Summary:**

We describe an unusual case of a male in his 30s with a history of neuroendocrine tumor (NET) who immigrated from Colombia and who underwent esophagogastroduodenoscopy (EGD) and colonoscopy for further evaluation of abdominal pain. He was incidentally found to have a female *Ascaris* worm in his stomach that was retrieved during the EGD.

**Conclusion:**

Despite its rarity, gastric ascariasis can cause various non-specific yet distressing gastrointestinal symptoms in patients, and therefore should be considered in those with risk factors for helminthic infections.

## INTRODUCTION

Ascariasis is an infection caused by the parasitic roundworm *Ascaris lumbricoides* (syn. *A. suum*), which is known to be one of the most common helminthic infections globally. It is spread by contaminated soil through the fecal–oral route and typically infects the small intestine, with rare reports of infection elsewhere in the body. Most reported infections are in areas of tropical or sub-tropical climates, such as Central African Republic, Chad, Ecuador, Indonesia, Liberia, Madagascar, and the Philippines, where soil can be contaminated with human or pig (the primary reservoir host) feces (1). In the United States, ascariasis cases associated with contamination with human feces have largely been eliminated due to infrastructure, and the disease is primarily zoonotic, associated with pig farming or using pig feces to fertilize vegetables (2). In the past 25 years, only 13 cases of gastric ascariasis have been reported in the literature (3). Only one case was reported in the United States in 2003 (4). Here, we present an unusual case of ascariasis found in the gastric body of a young adult male with a history of neuroendocrine tumor (NET), who presented with acute on chronic abdominal pain. It is important to distinguish gastric ascariasis from anisakiasis as the two nematode infections are managed differently.

## CASE PRESENTATION

A 37-year-old male with a neuroendocrine tumor (NET) presented to the emergency department with worsening generalized abdominal pain with associated nausea. In the emergency department, he underwent a CT of the abdomen that demonstrated known innumerable necrotic hepatic metastatic lesions up to 15.0 cm and hepatomegaly with mass effect. His abdominal pain was attributed to his malignancy and improved with supportive therapy. He was discharged home later that day.

**Fig 1:**
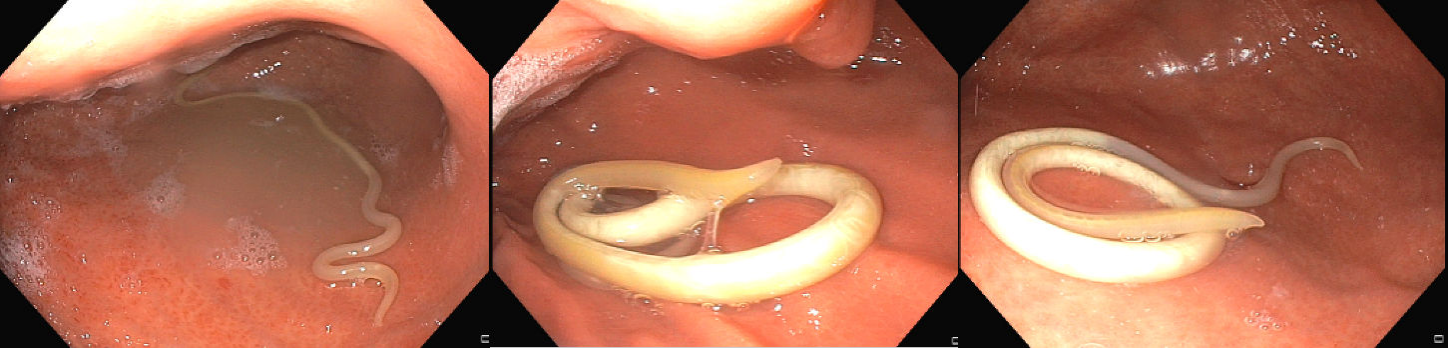
Endoscopic view of *A. lumbricoides*.

**Fig 2:**
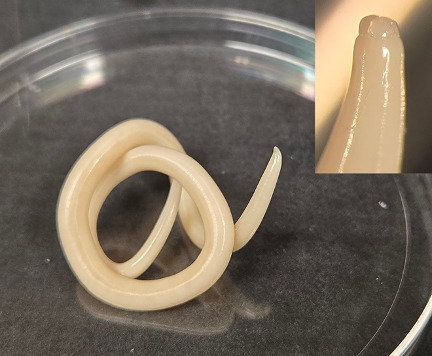
Retrieved *A. lumbricoides* on specimen dish. (Inset) Magnified view of oral orifice *of A. lumbricoides*.

Upon follow-up with his oncologist 1 week later, the patient reported continued abdominal pain for which he was then referred for esophagogastroduodenoscopy (EGD) and colonoscopy for further evaluation. The EGD and colonoscopy were largely unremarkable with the exception of a single worm that was found moving in the gastric body and promptly removed (Fig. 1). The live worm was sent in a sterile container to the laboratory for identification. The worm was later identified as *A. lumbricoides* based on its size (18 cm) and presence of three fleshy anterior “lips” (Fig. 2, inset). Laboratory microscopy revealed the worm, which had died at this point, to be a female *A. lumbricoides*, based on a relatively straight tail end lacking labial and caudal papillae and spicules. Upon further questioning, the patient reported that he had frequent travel between the United States, Central America, and Colombia within the prior year, as well as frequent contact with pigs in the United States through his work. Notably, he had normal eosinophil counts just prior to the EGD. Infectious Diseases service was consulted, and albendazole 400 mg and ivermectin 200 mcg/kg (single doses) were both started for ascariasis treatment, with instructions for close contacts to receive treatment as well. He subsequently followed up at the Infectious Disease Tropical Medicine Clinic and underwent a stool ova and parasite test, which came back negative.

## DISCUSSION

*A. lumbricoides* is a roundworm that infects over a billion individuals globally, mostly those living in Asia, Africa, and Latin America, where tropical and sub-tropical climates are prevalent (1). The humidity, warm temperatures, and poor sanitation support the development of the larvae in the soil. In the United States, immigrants from developing countries comprise a majority of individuals infected, although it is also a zoonotic disease in developed countries, with pigs serving as the primary reservoir (2).

In this case, we present a very rare occurrence of worm identification in the stomach and extraction via EGD. Only a handful of such occurrences are reported in the literature (5). *A. lumbricoides* infections are often diagnosed by visualization of worm expulsion through the stool, detection of eggs in concentrated wet mounts of stool, or by radiologic imaging. It is thought that the acidity and peristaltic movements of the stomach make it an unlikely location for *A. lumbricoides* to reside (6, 7). In our case, the presence in the stomach is likely incidental. And while the route to the stomach was not known, given the biology of the parasite, it probably migrated there as an adult worm from the small intestine. Ideally, these worms should be placed in formalin or alcohol and sent to the microbiology lab first for evaluation and documentation, after which they may be sent to the pathology lab for sectioning if macroscopic evaluation was not definitive.

A morphologic differential for *A. lumbricoides*, especially from the stomach, would be an anisakid nematode. Anisakids are similar in overall morphology and also possess three fleshy “lips,” but are almost never longer than 5.0 cm. Because they are L3 larvae in humans, they will always lack advanced sexual structures and eggs. Separating anisakids from *A. lumbricoides* is important because clinical management for the two diseases is different. In cases where gross morphology cannot definitively distinguish between the two genera, histologic analysis of transverse sections can aid in establishing a genus-level identification. In cases of anisakiasis, removal of the helminth is treatment, while ascariasis requires drug intervention.

Some of the previously reported gastric ascariasis cases were associated with new symptoms of hematemesis, gastric outlet obstruction, or anemia (5, 8). Our patient had gastrointestinal symptoms for 2 years prior to the diagnosis, which could have been attributable to his progressive NET. Thus, it is unclear when the patient acquired the helminthic infection and which symptoms were attributable to the ascariasis versus the NET. Given the patient’s history of travel to Central America and Colombia within the prior year, it is possible that the infection was acquired in one of these endemic areas; however, it is also possible that he contracted it in the United States given his close association with pigs at work. The prepatent period is approximately 2 months, and adult females can live for 12–18 months; therefore, simply the presence of an adult worm here was not helpful in determining where the patient was infected (9).

Common anti-helminthic treatments for ascariasis include albendazole, mebendazole, or pyrantel pamoate (10). Prognosis following treatment is often favorable.
